# Leveraging Artificial Intelligence to Predict and Manage Complications in Patients With Multimorbidity: A Literature Review

**DOI:** 10.7759/cureus.77758

**Published:** 2025-01-21

**Authors:** Sai Praneeth Chaparala, Kesha D Pathak, Rohit Rao Dugyala, Joel Thomas, Sai Prashanthi Varakala

**Affiliations:** 1 Internal Medicine, Gayatri Vidya Parishad Institute of Health Care and Medical Technology, Visakhapatnam, IND; 2 Medicine, Gujarat Adani Institute of Medical Sciences, Bhuj, IND; 3 Internal Medicine, Gandhi Medical College, Secunderabad, IND; 4 Internal Medicine, RAK Medical and Health Sciences University, Ras Al Khaimah, ARE; 5 Internal Medicine, Osmania Medical College, Hyderabad, IND

**Keywords:** artificial intelligence (ai), healthcare, machine learning (ml), medical imaging, multi-morbidity, predictive analysis

## Abstract

Artificial intelligence (AI) is revolutionizing healthcare by improving diagnostic accuracy, streamlining treatment protocols, and augmenting patient care, especially in the management of multimorbidity. This review assesses the applications of AI in forecasting and controlling problems in multimorbid patients, emphasizing predictive analytics, real-time data integration, and enhancements in diagnostics. Utilizing extensive datasets from electronic health records and medical imaging, AI models facilitate early complication prediction and prompt therapies in diseases such as cancer, cardiovascular disorders, and diabetes. Notable developments encompass AI systems for the diagnosis of lung and breast cancer, markedly decreasing false positives and minimizing superfluous follow-ups. A comprehensive literature search was performed via PubMed and Google Scholar, applying Boolean logic with keywords such as "artificial intelligence", "multimorbidity", "predictive analytics", "machine learning", and "diagnosis". Articles published in English from January 2010 to December 2024, encompassing original research, systematic reviews, and meta-analyses regarding the use of AI in managing multimorbidity and healthcare decision-making, were included. Studies not pertinent to therapeutic applications, devoid of outcome measurements, or restricted to editorials were discarded. This review emphasizes AI's capacity to augment diagnostic precision and boost clinical results while also identifying substantial hurdles, including data bias, ethical issues, and the necessity for rigorous validation and longitudinal research to guarantee sustainable integration in clinical environments. This review's limitations encompass the possible exclusion of pertinent studies due to language and publication year constraints, as well as the disregard for grey literature, potentially constraining the comprehensiveness of the findings.

## Introduction and background

**Figure 1:**
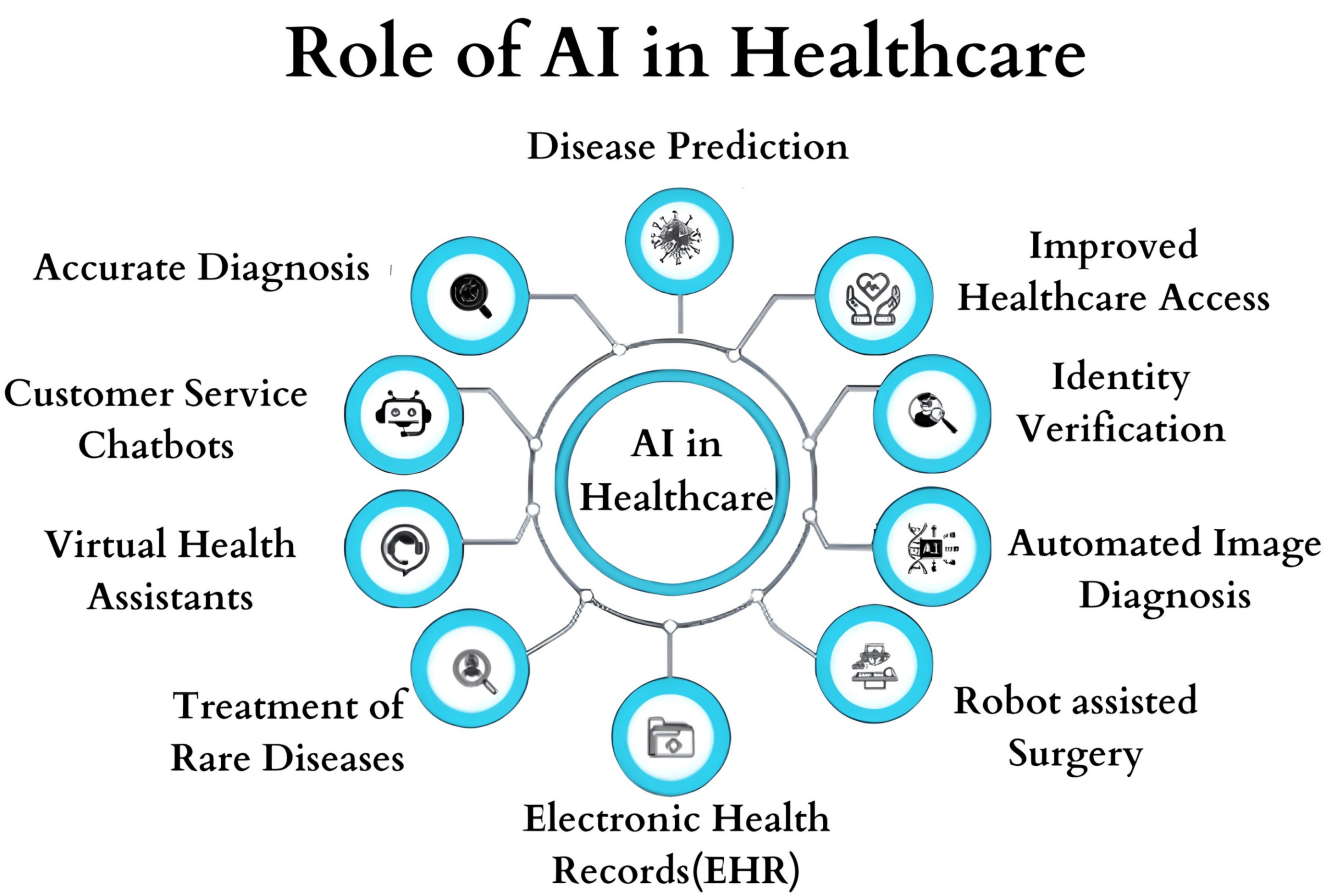
This image depicts the use of artificial intelligence (AI) in different fields of healthcare in providing better and enhanced care to patients. This image has been created by Rohit Rao Dugyala.

Artificial intelligence (AI) refers to the field of computer science focused on creating systems capable of performing tasks typically requiring human intelligence, such as problem-solving, learning, understanding natural language, recognizing patterns, and making decisions (Figure 1). AI spans from simple algorithms automating routine tasks to complex systems, like machine learning models and neural networks, that evolve and adapt over time [1]. Initially introduced in the mid-to-late 20th century, AI's role in medicine has significantly expanded in recent decades [1]. Early AI applications included basic expert systems like "MYCIN," designed for treating blood infections, and by the late 1900s, AI was applied to medical imaging, diagnostics, and decision-support systems. Advances in machine learning and data analytics in the early 21st century led to more sophisticated applications, such as improved diagnostic tools, predictive analytics, and personalized medicine [2]. Today, AI integration in medicine has accelerated, especially with the development of advanced algorithms, deep learning, and large-scale data analysis. AI is now integral to medical imaging, drug development, personalized treatment plans for multimorbid conditions, and patient management [3].

This study investigates the role of AI in predicting and managing complications in patients with multimorbidity, emphasizing its applications in improving diagnostic accuracy, treatment strategies, and patient outcomes. The literature review systematically examines relevant studies, focusing on AI’s use in predictive analytics and real-time data integration, particularly its ability to analyze large datasets from electronic health records (EHRs) and medical imaging. AI-driven models facilitate early prediction of complications, enabling timely interventions that enhance care in diseases such as cancer, cardiovascular disease, and diabetes. However, challenges remain, including the lack of longitudinal studies to evaluate AI's long-term impact, and biases resulting from insufficiently diverse training data. Therefore, collaboration among clinicians, data scientists, and policymakers is essential for the development of equitable and clinically relevant AI models. Regulatory frameworks, such as the FDA’s Digital Health Innovation Action Plan, play a vital role in ensuring the safe integration of AI technologies into healthcare.

## Review

Applications of AI in managing multimorbidity

**Figure 2:**
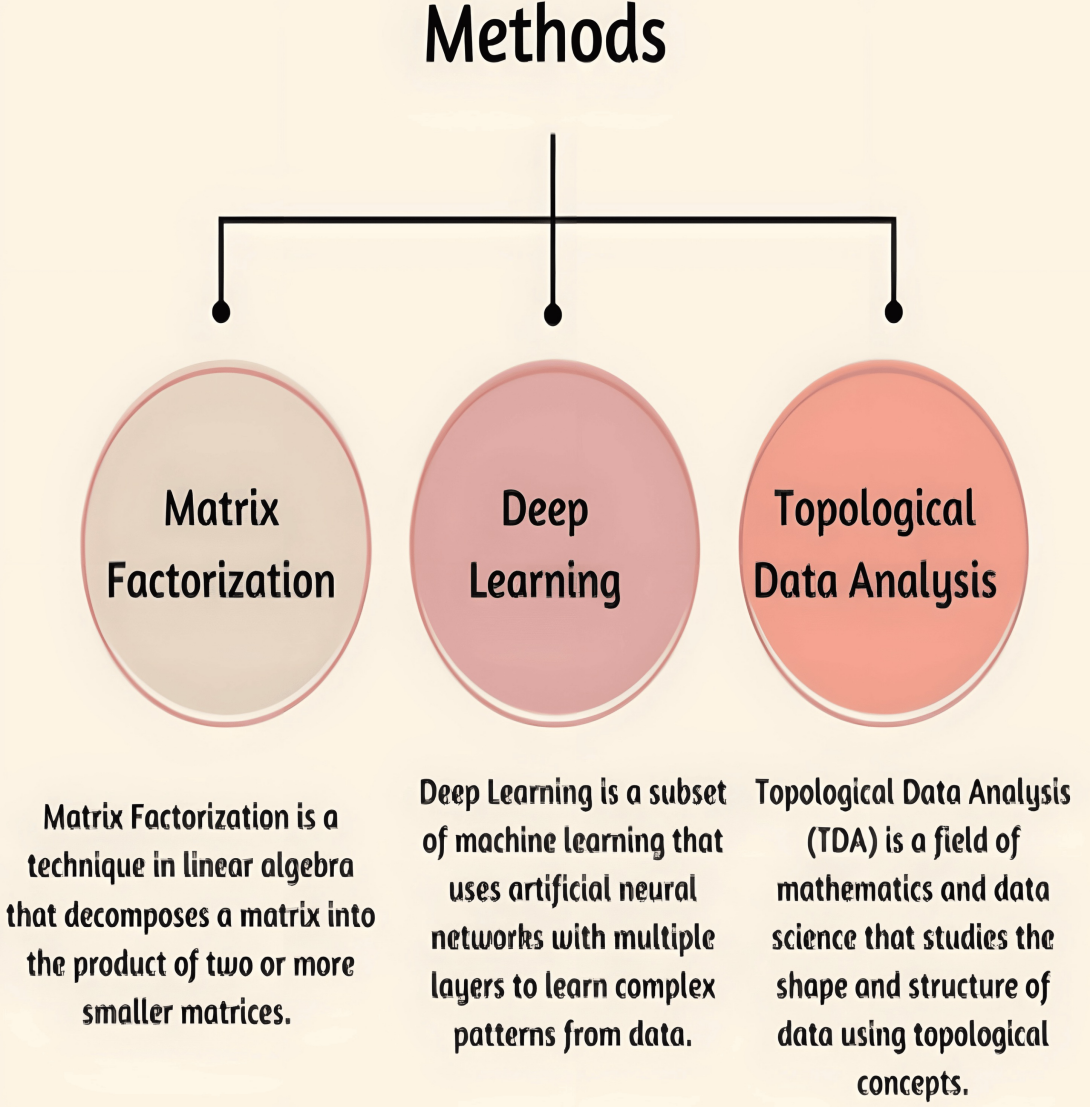
Advanced artificial intelligence (AI) methods in managing multimorbidity. This image has been created by Rohit Rao Dugyala.

Recent advances in medicine have led to an increase in life expectancy and a reduction in the incidence of major disabilities. This has also paved the way for an increase in chronic conditions (which are more prevalent in older ages) and their co-occurrence, known as multimorbidity [1]. There have been many advances in methods, shown in Figure 2, such as matrix factorization, deep learning, and topological data analysis, and how these can be used in multimorbidity research studies beyond cross-sectional, expert-driven, or confirmatory approaches to gain a broader and more advanced perspective of the evolving dynamics of multimorbidity treatment approaches and their understanding [1]. Multimorbid conditions present a significant challenge for healthcare professionals, impacting the cost-effectiveness and effectiveness of diagnosis and treatment of patients. Coexisting health conditions are prevalent among the aging population, which is expanding worldwide, while the average age of onset for these diseases is declining, leading to a rise in prevalence [1].

AI can be aimed at assisting healthcare professionals in addressing many challenges as it offers advanced analysis and point-of-care decision-making such as disease prediction, clinical strategies, identity verification, automated image diagnosis, robot-assisted surgeries, maintenance of EHRs, and virtual health assistants. Ground-breaking advances have been developed for predicting the length of stay of elderly patients with chronic diseases at the time of admission by combining network analytics and machine learning (ML). In a study by Hu et al. (2022), two networks were constructed: a multimorbidity network (MN) and a patient similarity network (PSN). The results of this experiment demonstrated that the network features could significantly enhance the performance of prediction models, providing valuable insights and prompting further research in the field of predictive analytics [2]. AI-based predictions help in providing specific treatment plans to each individual, such as determining the best option of multiple medications and identifying co-morbidities that were not previously recognized to be a risk for particular co-morbidities [3]. Training of healthcare professionals also paves the way for the development of telemedicine, which helps in the better delivery of health services to people in rural areas who face difficulties in accessing health services due to poor infrastructure. There are pilot projects that are thriving to build the foundation for the development of telemedicine, establish its effectiveness, and prove its necessity [4].

AI could be utilized for precision diagnostics in radiology. Magnetic resonance imaging (MRI) faces several significant challenges, including the necessity for expedited acquisition, improved workflow, and increased patient throughput, which are essential for making MRI widely accessible and affordable for all individuals. Furthermore, adapting to varying demographic patterns, customizing relevant protocols and procedures for an aging and multimorbid patient population, and delivering quantifiable data, reproducible imaging biomarkers, and integrated AI algorithms are crucial for establishing MRI as a fundamental component of contemporary precision medicine [5].

All these problems can easily be tackled by the proper and methodical application of AI to existing technology available for MRI scanning [5]. There has been a significant rise in interest and investment in AI technologies aimed at increasing their diagnostic accuracy, efficacy in treatment, and administrative output. All these problems can easily be tackled by the proper and methodical application of AI to existing technology available for MRI scanning [5]. There has been a significant rise in interest and investment in AI technologies aimed at increasing their diagnostic accuracy, efficacy in treatment, and administrative output. Many promising advances have been made in this field, including automated image analysis, improved imaging techniques, predictive analytics, radiomics and biomarkers, integration with EHRs, workflow optimization, and AI-powered decision support [2,3].

Predictive analytics - How AI can be used to predict complications

AI is revolutionizing medical care by predicting complications through advanced data analysis and predictive modeling. ML algorithms analyze EHRs to identify risk factors leading to complications. AI tools can also analyze medical images, detecting abnormalities and disease development before they become clinically significant. AI-driven predictive analytics is used for effective patient management, integrating data from wearable health devices, pulse oximeters, and blood pressure machines. This helps reduce hospital readmissions in high-risk patients, especially those with chronic obstructive pulmonary disease, heart failure, diabetes, and septicemia, and aids in research on new treatments [6].

Case Studies and Examples

Lung cancer is the most prevalent cancer globally and the most frequent cancer in men. The identification of ground-glass nodules (GGNs) in lung imaging is essential for an improved, expedited, and precise diagnosis. Li et al.'s (2024) findings suggested the presence of both benign and malignant tumors. Clinicians face considerable challenges in appropriately identifying GGNs and selecting appropriate therapeutic options, which significantly impact patient outcomes. AI has demonstrated significant efficacy in the assessment of GGNs by processing imaging data, including CT scans, MRIs, and X-rays, while predicting the benign or malignant status, pathological subtypes, and genetic alterations of GGNs [6].

Integration of AI in Patient Management

ML has seen a rising trend in subtype definition and risk prediction, particularly in cardiovascular diseases. Banerjee et al. examined the utilization of ML for subtype classification and risk assessment in heart failure (HF), acute coronary syndromes (ACS), and atrial fibrillation (AF), revealing that ML lacked sufficient accuracy. The clinical utility depended on enhancements in development, validation, and impact, supported by a straightforward checklist. This study was constrained by the quantity and nature of the included covariates, ML methodologies, sample size, geographical location, clinical context, and emphasis on individual diseases [7].

Existing AI predictive models in healthcare have great potential for improving diagnostic accuracy and patient outcomes. However, their effectiveness depends on rigorous validation, high-quality data, and seamless integration into the clinical practice. Ongoing research and development are essential to address the existing limitations and enhance the utility of AI in healthcare.

Identifying the Lack of Extensive, Longitudinal Studies on AI’s Impact

**Figure 3:**
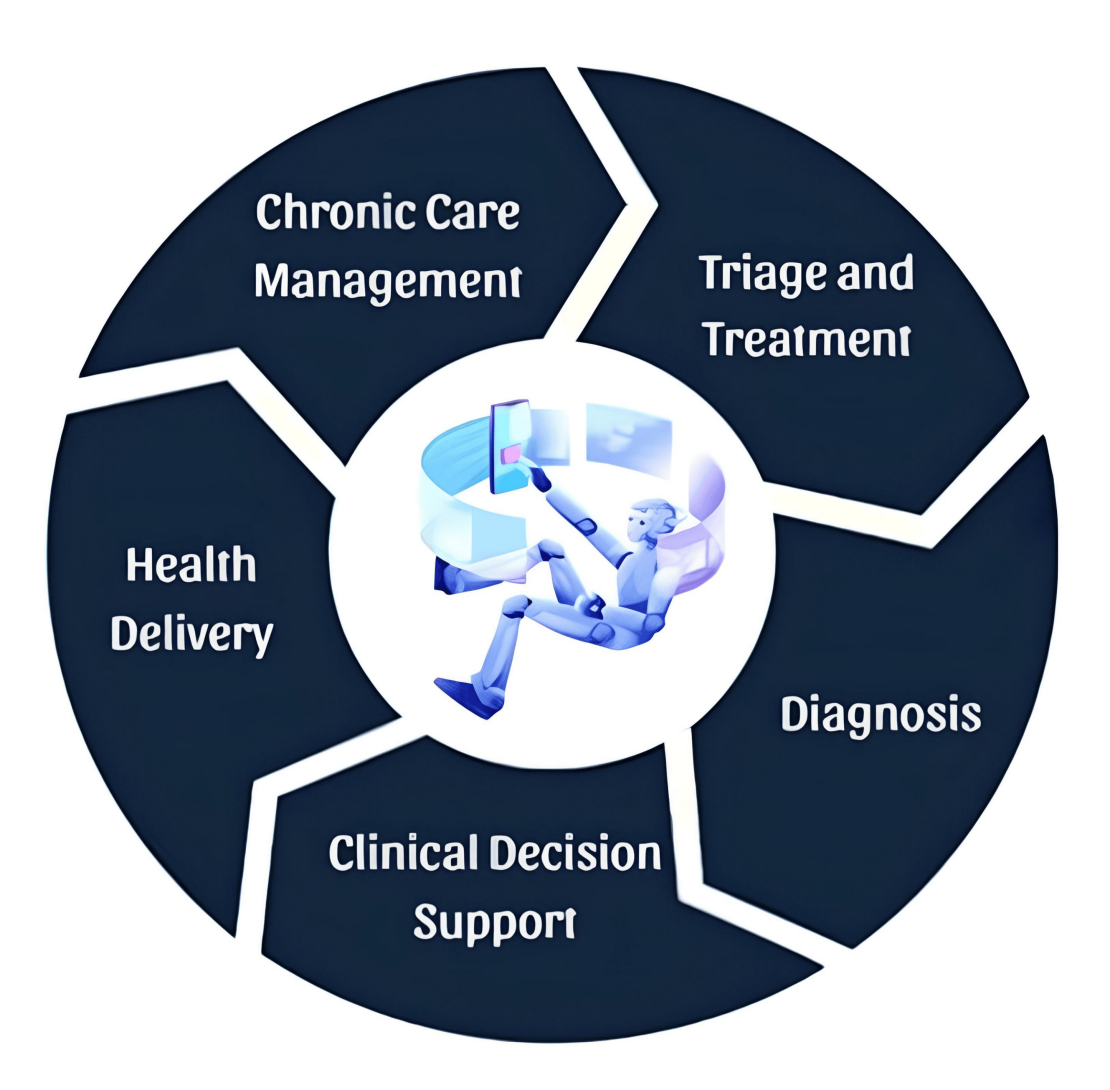
This image depicts the areas of artificial intelligence (AI) healthcare. This image has been created by Rohit Rao Dugyala.

Multi-morbid conditions are anticipated to exacerbate the lives of individuals impacted if no targeted intervention is executed. Research on capital expenditure and resource allocation concerning the utilization of AI for improved healthcare outcomes is on the rise [7]. Nonetheless, the majority of this research has concentrated on short-term or particular uses rather than on a broader and more extended range of usage. Longitudinal studies are essential for comprehending the sustained benefits, unexpected challenges, and their enduring impact on healthcare over time [8]. Moreover, alternative research methodologies are essential for a thorough assessment of AI in healthcare, including implementation research to examine AI-integrated service delivery, translational research to assess clinical decision-making and management, and precision diagnostics for evaluating long-term clinical outcomes [9]. Figure 3 illustrates the use of AI in healthcare.

Integration of AI into clinical practice

AI is transforming healthcare by enhancing diagnostic precision and decision-making procedures. AI systems can analyze extensive patient data and deliver immediate insights into individualized treatment strategies. This diminishes administrative burden, enabling healthcare providers to concentrate on patient care. AI-driven predictive analytics detects possible dangers and enhances resource allocation, resulting in better patient outcomes. AI-driven algorithms, especially deep learning models, analyze images like X-rays, MRIs, and CT scans with exceptional accuracy. AI also aids in pathology by analyzing tissue samples for cancerous cells, providing faster and more accurate results [6].

Personalized Treatment Plans With AI

AI plays a critical role in personalized medicine and in tailoring treatment plans based on individual data, leading to more effective therapies and fewer side effects. AI-powered wearable devices monitor vital signs and provide real-time alerts to patients and healthcare providers. AI-driven chatbots and virtual assistants support patient care by offering preliminary medical advice, managing appointments, and remotely monitoring chronic conditions [7]. In genomics, AI aids in analyzing genetic data, improving disease understanding, and enabling innovative treatments [8]. The widespread adoption of next-generation sequencing (NGS) in oncology has advanced individualized cancer treatment; however, many clinicians still struggle to translate molecular data into actionable insights. AI systems are increasingly being used to support decision-making in precision oncology, though challenges and limitations remain [9]. AI is transforming prostate cancer pathology by enhancing detection, grading, outcome prediction, and identification of molecular subtypes, offering a valuable collaboration with pathologists to reduce workload and aid in treatment planning [10]. AI models in reproductive medicine enable personalized, quantitative predictions of in vitro fertilization (IVF) success, offering precise success rates that enhance clinical planning and informed consent. AI-guided gonadotropin dosing personalizes treatment, showing early promise in improving outcomes, such as the number of mature oocytes and usable blastocysts, while also standardizing dosing across providers [9,10].

These AI tools not only optimize treatment but also hold the potential for cost savings by identifying the lowest effective dose based on personalized profiles. For example, AI-driven decision-support systems in oncology have demonstrated cost-effectiveness by reducing unnecessary diagnostic tests and streamlining treatment pathways without compromising patient outcomes in identifying precancerous lesions [11].

Studies show that AI-aided colonoscopies lead to higher adenoma and polyp detection rates, improving early cancer detection outcomes compared to routine colonoscopies [12]. Advanced AI applications are transforming nephrology by enhancing predictions and aiding treatment decisions. For instance, a model developed for end-stage kidney disease (ESKD) patients predicts imminent hospitalizations within seven days using over 1,500 variables, including treatment vitals and clinical notes, improving workflow for nurses and offering real-time insights into patient risk levels. In chronic kidney disease (CKD), AI models, such as random forests and artificial neural networks, have been used to predict disease progression and patient outcomes, with some models achieving over 95% accuracy in predicting estimated glomerular filtration rate (eGFR) at various intervals [13].

Moreover, the CKD Forecaster Tool, which incorporates AI-driven predictions, has been used in clinical settings to improve patient education and transition planning, resulting in fewer patients requiring hemodialysis with a central venous catheter. AI has also shown promise in optimizing drug prescriptions, such as erythropoietin dosing for anemia management in patients with ESKD, where it has reduced hemoglobin variability and erythropoietin dosage [13]. AI-based radiomics has significantly advanced the clinical management of hepatocellular carcinoma (HCC) by enhancing diagnosis, personalizing treatment, and improving survival prognosis [14]. AI has proven to be highly useful in the management and treatment of asthma by enhancing the classification of asthma phenotypes, which is crucial for personalized treatment. ML algorithms, such as latent class analysis, have been effectively used to differentiate asthma subtypes based on symptoms, genetic data, and response to treatment, leading to more tailored therapeutic strategies. AI models have also improved the prediction of asthma exacerbations and hospital admissions, enabling timely interventions to prevent severe outcomes.

Additionally, AI has facilitated the monitoring of asthma control levels, providing accurate assessments that can help physicians promptly adjust treatment plans. Moreover, AI systems have shown potential in predicting treatment efficacy for asthma, promising more effective and personalized care for patients [15]. AI offers significant advantages in managing AF by enhancing the precision of ablation procedures through the accurate identification of spatiotemporal activation patterns in intracardiac electrograms. It also aids in the clinical phenotyping of AF, allowing for more personalized treatment strategies and better prediction of patient outcomes. Furthermore, AI improves the prediction of long-term treatment efficacy and arrhythmia risk, facilitating more informed clinical decision-making and advancing personalized medicine in AF management. AI methods hold significant promise for enhancing the diagnosis and treatment of AF by enabling large-scale, accurate AF detection and guiding personalized treatment through advanced data analysis and virtual patient comparisons. AI will also revolutionize the interpretation of complex intracardiac recordings and imaging, uncovering hidden biomarkers and automating workflows for more efficient AF management [16]. AI has been applied in abdominal aortic aneurysm (AAA) management to enhance image segmentation, allowing for precise quantitative analysis and characterization of AAA morphology, geometry, and fluid dynamics.

By processing large datasets, AI identifies patterns predictive of AAA growth and rupture, aiding risk assessment. Predictive models developed with AI assess postoperative outcomes, including mortality and complications, following endovascular aneurysm repair. These tools support surgeons in preoperative planning, optimizing surgical decisions, and developing personalized treatment strategies [17]. AI in clinical practice has tremendous potential, but several significant limitations still impede its widespread adoption.

AI has limitations in various aspects of clinical care. This may not always accurately represent a patient's clinical context, such as infection-induced insulin resistance. In addition, AI tools trained on biased datasets may misdiagnose symptoms or downplay risks in underrepresented populations, leading to suboptimal care. Additionally, AI may not fully understand the emotional and ethical aspects of clinical care, such as quality-of-life concerns [17].

Interoperability and Data Utilization as per the Healthcare Information and Management Systems Society (HIMSS) Analytics

According to a report published in 2023, 97% of healthcare data will be unused because they are unstructured, posing a significant challenge to AI integration. ML and natural language processing (NLP) tools are being developed to address this; however, the gap in structured data remains a critical barrier to fully realizing AI’s potential of AI in healthcare systems.

Training and workforce implications as per HIMSS analytics: Studies highlight that healthcare professionals often lack sufficient training in AI tools, which creates reluctance and hesitancy in adopting these technologies. This limitation not only affects the efficient use of AI but also raises concerns about errors or misapplication of AI-generated insights.

Patient safety concerns: The Journal of the American Medical Association (JAMA) emphasizes the evidence gap in demonstrating AI’s real-world effectiveness of AI, especially regarding patient safety. Research is still ongoing to assess whether AI-driven interventions truly enhance clinical outcomes without introducing new risks [18].

Bias and equity: There are ongoing concerns about AI algorithms perpetuating healthcare inequities. Studies point to the biased nature of AI models trained on data that are not representative of diverse populations, potentially exacerbating health disparities [18].

Global challenges: In low-resource settings, the integration of AI faces additional hurdles, such as limited infrastructure and technological capabilities. AI solutions must adapt to these environments to have a meaningful global impact; however, this is still an underdeveloped area [18].

Ethical and legal issues surrounding AI in healthcare

AI has shown potential in the healthcare sector, especially when it comes to managing patients with multimorbidity. AI can increase predictive accuracy, optimize treatment plans, and improve patient outcomes. AI in healthcare is directly associated with individual's health data, which leads to legal and ethical issues that include inaccuracy, privacy, medical errors, bias, commercialization of data, lack of emotional viewpoints, and liability.

Concerns about newer digital technologies becoming a new source of inaccuracy and data breaches have arisen as a result of its use [19]. For instance, AI technologies may produce inaccurate results that discriminate against particular patient groups owing to their reliance on faulty or biased datasets. Gerdes et al. (2024) point out that if an AI tool used in dermatology is trained on pictures of people with primarily fair skin tones, it would lead to an underdiagnosis of other skin types [20].

AI technologies can also reinforce health disparities, as shown by Obermeyer et al. (2019) [21], where a US AI tool incorrectly gave an excessively low-risk score to patient groups with dark skin types, even though these patients were clearly sicker than identical patients with light skin types. The reliance of this algorithm on healthcare cost prediction rather than disease diagnosis is the source of this issue.

Privacy is another issue that influences AI development and testing. The creation of AI applications may necessitate revisions to privacy and confidentiality rules and regulations. To share health information with any third party who is not directly involved in the patient's treatment, it is necessary to obtain the express agreement of the patient in the United Kingdom. Without patient consent, researchers must obtain permission from the Confidentiality Advisory Group (CAG) of the Health Research Authority to access private patient data [22]. Lack of access control and failure to manage user access to patient data properly can also lead to data breaches where hackers or malicious actors gain unauthorized access to patient data (such as medical records or insurance information), potentially causing significant financial and reputational damage to healthcare providers [23].

The potential for medical errors in prescriptions is also a concern. If a doctor prescribes medication using adult doses for children, and the AI does not have a guideline to spot the error, it can lead to deadly adverse effects. The same can happen when AI provides an incorrect diagnosis, which can lead to incorrect treatment. This happens because AI learns from already published data [24].

Another prominent issue is discrimination and bias toward specific groups based on the dataset used for learning. The narrow representation of training data for a specific demographic can lead to biases when the AI system is used outside the demographic. Similarly, an AI system trained on data obtained from high-level medical facilities may exhibit biases when used in lower-level medical facilities [25].

Commercialization of biospecimens and patient data is another problem. Whether it is appropriate for researchers or businesses to make money out of these data without providing the patients with all the information is a matter of ethics. Such uses are allowed under current legislation, such as the Common Rule, but only if the patients are informed beforehand. Despite this, patients' mistrust of commercial entities is frequently not sufficiently addressed. The ethical and legal issues associated with the malpractice and bias of AI algorithms stem from the possibility that these tools would reinforce errors or biases found in the training data, which could endanger patients [26].

Additionally, the lack of emotional and human viewpoints necessary for healthcare delivery and research accompanies AI applications in healthcare. It is also crucial to address any potential psychological, financial, and social repercussions of applying large language models (LLM) tools in healthcare settings. Undervaluing the function of the human brain is a problem that cannot be ignored. To this end, highlighting the vital role that humans play in healthcare practices and research is essential [27].

The liability and accountability for the use of AI applications is another impending legal concern. There are still unanswered questions about who bears the ultimate responsibility for decisions regarding patient care made using or influenced by AI applications. Should medical professionals be held solely accountable for choices made by algorithms that they do not fully comprehend? Doctors will employ a system that they do not comprehend. Is the developer liable for anything? The rationale behind an AI application is challenging and frequently too sophisticated to explain, making the problem difficult. As AI systems are "learning" from data, they constantly adapt and evolve in unpredictable ways [28].

Policies and Guidelines

AI systems must uphold and defend general ethical standards. The requirement for interdisciplinary collaboration involving humanistic as well as technical, social, and healthcare competencies presents the largest obstacle to value-based design approaches and ethics based on design principles. A bridge-building function can be included in various system development scenarios to help reconcile a multitude of disparate needs and considerations. However, clinical domain experts must be involved in the development of AI systems in healthcare settings.

It is also important for patients to be included in the AI development process. Their viewpoints can aid in identifying potential moral dilemmas and supporting the development and proper application of AI in healthcare. When patients are involved, their needs can be better served, and public confidence in research can increase [29].

There are various standards with additional AI recommendations for clinical study documentation for diagnostic accuracy, reporting randomized trials, and medical imaging. The Standards for Reporting of Diagnostic Accuracy Studies (STARD) 2015 statement remains the most widely accepted set of reporting standards for diagnostic test accuracy studies. The STARD was developed to improve the completeness and transparency of studies investigating diagnostic test accuracy. It consists of a checklist of 30 items that the authors are strongly encouraged to address when reporting diagnostic test accuracy studies. It has been endorsed by over 200 biomedical journals, and studies have shown that adherence to the STARD checklist leads to improved reporting of key study parameters. The goal of the STARD-AI protocol is to establish a thorough reporting process for investigations of the accuracy of diagnostic tests centered on AI. The reporting of AI-driven diagnostic tools must be more transparent and clearer, and this guideline is crucial for achieving this goal. It fills in the blanks in conventional reporting frameworks, notably the lack of information on AI-specific procedures, such as model validation, data pretreatment, and fairness measures. Recommendations for reporting techniques, such as model development decisions, human-AI interactions, and external validation, are included in the STARD-AI. Additionally, it places a strong emphasis on transparency when presenting results, which facilitates stakeholder comparison of AI diagnostic models and promotes the consistent application of AI in clinical contexts [30].

The Consolidated Standards of Reporting Trials (CONSORT) statement provides evidence-based recommendations to improve the completeness of reporting randomized controlled trials (RCTs). This statement was first introduced in 1996 and has since been widely endorsed by medical journals internationally [31]. Comprehensive reporting guidelines for clinical trials involving AI-based therapies are provided via the Consolidated Standards of Reporting Trials-Artificial Intelligence (CONSORT-AI) extension [32]. It expands on the baseline CONSORT 2010 principles by introducing 14 new AI-specific elements. These include the requirement to provide a detailed description of the creation of the AI intervention, data processing methods for input and output, communication between AI and human users, and error analysis. Guaranteeing transparency and reproducibility enhances the dependability and credibility of AI solutions in healthcare environments. Additionally, there are examples that help implement these standards. For ethical guidelines, refer to Table 1 [33].

**Table 1:** This table outlines the moral standards that artificial intelligence (AI) systems ought to follow. This table has been created by the author.

Ethical principles	Description
Respect for human agency	It is important to recognize that people have the right to make their own decisions and take independent action. Three additional specific principles—autonomy, dignity, and freedom—that characterize fundamental human rights are encapsulated in the concept of respect for human agency [33].
Data governance and privacy	Individuals have the right to data protection and privacy, which should always be upheld [33].
Fairness	No one should be unfairly benefited or disadvantaged; everyone should have equal rights and opportunities [33].
Individual, social, and environmental well-being	Every system ought to promote these three areas of well-being rather than undermine them [33].
Transparency	All parties involved in Al programs should be aware of and able to comprehend the programs' goals, inputs, and workings [33].
Accountability and oversight	People should be able to comprehend, oversee, and regulate the architecture and functioning of Al-based systems, and those engaged in their creation or management ought to bear accountability for the outcomes that arise from how these applications perform [33].

MAS-AI (Model for Assessing the Value of Artificial Intelligence in Medical Imaging) [34] was created to assess the use of AI in medical imaging. It employed a two-step procedure that addressed five process elements and nine domains.

Step 1: Concentrate on the traits of the patient, the creation of the AI model, and important moral and legal issues.

Step 2: Entail a multidisciplinary assessment of the results in five areas: clinical impact, safety, economics, organizational considerations, and patient aspects.

For healthcare stakeholders, this paradigm guarantees evidence-based AI adoption in medical imaging while offering transparency and organized decision-making.

Prevalent techniques include the expectation that algorithms may be examined, even though safeguards to guarantee that AI applications are secure and efficient are still being developed. "The output of each algorithm should be explicable.” Developers must be able to create the algorithm for examination, provide evidence for the algorithm's effectiveness, and guarantee that the program can deliver the desired results via testing or certification procedures to promote the adoption and acceptance of AI applications. Product master data, which contain information about the individual parts of the product, may also contain details about the algorithm used. In the future, the algorithm that was used to validate or authenticate healthcare decisions made using patient data may be included in specific health information. Furthermore, there may be a need to audit AI events for reporting purposes [28].

Future Directions for Intervention in Multimorbidity Management

Although AI has the ability to completely transform the way multimorbidity is managed, there is still a large research gap in the creation of thorough frameworks that would govern its moral use. Although AI can improve patients' tailored care and forecast accuracy for numerous chronic illnesses, its implementation raises questions regarding algorithmic bias, data privacy, and transparency. Currently, studies tend to concentrate more on the technical aspects of AI than on the moral ramifications of using it in intricate healthcare situations. To ensure that AI applications in multimorbidity management are in line with the values of justice, responsibility, and patient autonomy, frameworks that address these ethical issues are required. AI can also perform background data analysis to enable providers to have a more integrated record of their patients during consultation. AI may also play an important role in identifying at-risk patient populations. AI systems may help reduce health inequalities by surfacing the most vulnerable patients [24]. Additionally, these frameworks must consider the potential socioeconomic gaps caused by the use of AI, guaranteeing fair access to AI-driven healthcare.

Future directions and recommendations

AI plays an essential role in healthcare by assisting physicians with diagnosis and treatment. Studies have shown that AI can work faster and more accurately than doctors. For example, AI in EHRs can reduce manual entry errors, improve patient record quality, and save time. Recent models also predict complications and aid in decision-making. However, more research is needed to expand the datasets, improve neural networks, and validate AI performance in real-world scenarios [35].

AI has demonstrated high diagnostic accuracy in areas such as lung cancer, breast cancer, and neurological conditions using image analysis; however, there remains a gap between controlled settings and everyday clinical effectiveness. Bridging this gap will require further research, and the success of validation relies on collaboration among clinicians, data scientists, and policymakers, focusing on strong, clinically useful models.

The lack of AI training data, particularly regarding ethnicity, sex, and socioeconomic factors, presents a challenge. Addressing these biases to ensure that AI serves all populations fairly is a key research goal [36].

The FDA’s Digital Health Action Plan and Software Precertification Program provide expedited pathways for AI technologies classified as software as a medical device (SaMD), ensuring safety assessments without stifling innovation. Global initiatives such as the International Medical Device Regulators Forum (IMDRF) aim to standardize regulations across nations to ensure the safe integration of AI in healthcare [37].

Recommendations

Although hospitals are aware of patient information management, AI-driven storage solutions are now employed to store and protect personal data, including sensitive genomic data. Advanced encryption techniques and cybersecurity measures are necessary to safeguard these data, with de-identification helping minimize breaches [38-40].

Interdisciplinary collaborations interpret complex images and improve the workflow and diagnostic accuracy. AI-based algorithms perform well in segmenting lung nodules, distinguishing between benign and malignant lesions, and reducing the need for unnecessary follow-up scans. This technology could prove invaluable for noninvasive tumor characterization and mutation prediction [41,42]. In laboratory diagnostics, cell morphology has the potential to increase accuracy and reduce missed diagnoses, making tedious manual microscopy tasks more efficient [43].

Patient-Centric Approaches

While AI offers transformative capabilities in diagnostics and data analysis, it is vital to recognize that certain human elements in patient care, such as empathy, communication, and a personalized approach, remain irreplaceable. Effective history-taking and patient communication are essential for understanding a patient’s condition holistically. AI cannot replicate these human qualities but can complement them by automating routine, time-intensive diagnostic tasks, thereby allowing physicians to dedicate more time to meaningful doctor-patient interactions [44].

The integration of AI into clinical workflows should focus on enhancing patient-centered care rather than detracting from it. By streamlining repetitive processes, AI has the potential to improve the doctor-patient relationship, enabling physicians to focus on providing empathetic, individualized care. Achieving this balance requires thoughtful implementation strategies that embed AI tools into healthcare practices without compromising care quality [44].

To maximize the benefits of AI while preserving the human aspects of healthcare, continuous training for healthcare providers is crucial. This training should emphasize the effective use of AI tools and their implications for patient relationships. Moreover, regular monitoring of AI’s impact on doctor-patient interactions will ensure that time saved through automation is reinvested in fostering stronger, more empathetic connections with patients [44].

By establishing clear guidelines on AI use, prioritizing patient-centered approaches, and maintaining a focus on empathy, AI can truly complement the physician’s role in building trust and delivering holistic care.

Educational Initiatives and Global Health

AI-driven systems can now assist nurses with documentation, reducing time spent remotely away from the patients. Hospital Pressure Ulcer Manager has reduced the incidence of complications and ICU stays. The example provided aligns with the theme of nurse time-saving by highlighting how AI-driven systems can assist in reducing the administrative burden, such as documentation tasks, allowing nurses to spend more time directly with patients. The Hospital Pressure Ulcer Manager is an example of how AI can reduce complications and ICU stays, thereby improving patient outcomes and reducing the need for extensive manual monitoring. By automating routine tasks and enhancing efficiency, AI enables nurses to focus on higher-priority clinical activities. Additionally, AI's role in resource-poor settings, including emergency management and disease prediction, further supports time-saving by streamlining decision-making and improving the timely delivery of care in critical situations. This integration of AI in nursing workflows ultimately contributes to more efficient use of time, ensuring that nurses can provide better, more focused care to their patients. Additionally, AI shows promise in resource-poor settings, helping identify and treat emergencies such as birth asphyxia, predicting disease outbreaks, and providing therapeutic recommendations [45,46].

AI is shifting healthcare worldwide, especially in resource-limited situations. AI has addressed special issues in low- and middle-income countries (LMICs) with limited healthcare experts, weak infrastructure, and high disease burdens. AI-powered mobile health apps and telemedicine platforms are boosting healthcare access in underserved areas by providing remote consultations and health education. Additionally, cost-effective AI-driven diagnostic methods like automated image analysis for tuberculosis diagnosis and malaria screening have shown promise in improving early detection and treatment. Despite these advances, LMICs still struggle to implement AI due to data quality, digital illiteracy, and infrastructure issues [46].

Research gaps

Despite advancements in healthcare technology, several critical gaps have hindered the effective integration of AI and longitudinal studies into clinical practice. Longitudinal studies are crucial for understanding patient outcomes and healthcare delivery efficiency. However, short-term studies may not capture long-term data [47]. Rapid technological advancements in healthcare pose challenges, as innovations can become outdated quickly [47]. Data quality is crucial for AI models' effectiveness. Successful AI implementation requires ease of use and enhanced decision-making [47]. There is a growing literature on AI and ML applications in cardiovascular medicine, but further refinement is needed to fully realize their potential [47]. Continuous updates and retraining are necessary for accuracy. Personalized treatment approaches for comorbid conditions are essential (Tables 2, 3) [48].

**Table 2:** Applications of AI in disease detection and risk prediction. This table has been created by the author. AI: artificial intelligence; ML: machine learning; GGNs: ground-glass nodules; CVS: cardiovascular system.

Strategy	Description	Key AI technologies	Benefits	Challenges
Predictive analytics in lung cancer	Detection of GGNs is a cornerstone in the early detection of lung cancer	Deep learning algorithms	Early detection; better prognosis; timely intervention	Extensive validation studies; standardization of imaging protocols; and improving the interpretability of AI algorithms [6].
Subtype definition and risk prediction	Use of AI in subtype definition and risk prediction in cardiovascular diseases	Machine learning	Better diagnosis and effective treatment	Number and type of included covariates, ML methods, population size, country, clinical setting, and focus on single diseases [7].
AI and ML in CVS	Overview of AI and ML as it relates to cardiovascular healthcare	Machine learning	Automated imaging interpretation; automated data extraction and quality control; clinical risk prediction	Further refinement and evaluation [48].

**Table 3:** AI applications in clinical prediction and decision-making across medical conditions. This table has been created by the author. AI: artificial intelligence; ML: machine learning; RCT: randomized controlled trial; TKA: total knee arthroplasty; AKI: acute kidney injury; EHR: electronic health records.

AI	Intervention condition(s)	Addressed study design (e.g., case study and RCT)	Outcomes	Limitations
Applied informatics and predictive modeling	Prediction of opioid use disorder	Retrospective cohort study	Compared to prior models is more accurate and reliable in predicting patients with opioid use disorder	Under-reporting of opioid issues reduced model generalizability [49].
Boosted ensemble machine learning	Predicts adverse cardiac events by analyzing CT scans	Literature review	Higher prognostic accuracy for mortality rates	Selection bias and inter-rater variability [50].
AI applications in renal disease	Image classification of arteriovenous fistula aneurysm	Narrative review	Convolutional neural network model classified arteriovenous fistula aneurysm with >90% accuracy	Need for advanced computational resources [13].
Machine learning, deep learning, natural language processing	Polytrauma patient management	Systematic review	Accurate and reliable predictions in polytrauma patients thus facilitating timely interventions	Heterogeneity of subjects [51].
Machine learning, deep learning, deep neural network	Development of anticancer drugs	Literature review	Predicts the sensitivity of cancer cell lines to different drugs and accelerates drug development	Deep learning models are difficult to interpret [52].
ML methods like random forest and gradient boosting machine	Total knee arthroplasty: predicts TKA component size and postoperative complications	Systematic review	Predicts complications like AKI and patient satisfaction after TKA	The study cannot be generalized because of a small cohort of patients and reliance on EHR, which lacked detailed clinical information [53].
Random forest machine learning AI	Distant metastasis in colorectal cancer patients	Cohort study	Surgery, chemotherapy, and radiotherapy were required to improve prognosis in high-risk patients while low-risk patients benefit only from surgery and chemotherapy	Random forest models are difficult to interpret [54].

## Conclusions

AI integration in healthcare offers transformative potential, yet significant barriers must be addressed to harness its full capabilities. This paper underscores the necessity for longitudinal studies to thoroughly assess AI’s effectiveness over time, especially concerning the management of comorbid conditions where treatment complexity increases. Ethical issues related to data privacy, algorithmic bias, and accountability require urgent attention to promote responsible AI usage. Additionally, involving patients in the development of AI tools and enhancing training for healthcare professionals on these technologies are essential steps for successful implementation. By fostering interdisciplinary collaboration, establishing comprehensive regulatory frameworks, and prioritizing the elimination of biases in AI training data, the healthcare sector can leverage AI to significantly improve diagnostic capabilities and treatment outcomes. Ultimately, this approach will advance healthcare delivery and contribute to more equitable and effective patient care across diverse populations, addressing complex medical conditions and enhancing overall health outcomes.
